# What proteins and albumins in bronchoalveolar lavage fluid and serum could tell us in COVID-19 and influenza acute respiratory distress syndrome on mechanical ventilation patient - A prospective double center study

**DOI:** 10.2478/jccm-2025-0005

**Published:** 2025-01-31

**Authors:** Anita Djurdjevic Svraka, Dragan Svraka, Bosa Mrjanic Azaric, Jovana Malic, Goran Baric, Pedja Kovacevic

**Affiliations:** Faculty of Medicine, University of Banja Luka, Banjaluka, Bosnia and Herzegovina; University Clinical Centre of the Republic of Srpska

**Keywords:** acute respiratory distress syndrome, mechanical ventilation, COVID-19, influenza type A

## Abstract

**Introduction:**

The extent of in vivo damage to the alveolar-capillary membrane in patients with primary lung injury remains unclear. In cases of ARDS related to COVID-19 and Influenza type A, the complexity of the damage increases further, as viral pneumonia cannot currently be treated with a causal approach.

**Aims of the study:**

Our primary goal is to enhance the understanding of Acute Respiratory Distress Syndrome (ARDS) by demonstrating damage to the alveocapillary membrane in critically ill patients with COVID-19 and influenza type A. We will achieve this by measuring the levels of proteins and albumin in bronchoalveolar fluid (BAL) and serum. Our secondary objective is to assess patient outcomes related to elevated protein and albumin levels in both BAL and blood serum, which will deepen our understanding of this complex condition.

**Materials and methods:**

Bronchoalveolar lavage (BAL) fluid and serum samples were meticulously collected from a total of 64 patients, categorized into three distinct groups: 30 patients diagnosed with COVID-19-related acute respiratory distress syndrome (ARDS), 14 patients with influenza type A (H1N1 strain), also experiencing ARDS, and a control group consisting of 20 patients who were preoperatively prepared for elective surgical procedures without any diagnosed lung disease. The careful selection and categorization of patients ensure the robustness of our study. BAL samples were taken within the first 24 hours following the commencement of invasive mechanical ventilation in the intensive care unit, alongside measurements of serum albumin levels. In the control group, BAL and serum samples were collected after the induction of general endotracheal anaesthesia.

**Results:**

Patients in the COVID-19 group are significantly older than those in the Influenza type A (H1N1) group, with median ages of 72.5 years and 62 years, respectively (p < 0.01, Mann-Whitney U test). Furthermore, serum albumin levels (measured in g/L) revealed significant differences across all three groups in the overall sample, yielding a p-value of less than 0.01 according to ANOVA. In terms of treatment outcomes, serum albumin levels also exhibited a significant correlation, with a p-value of 0.03 (Mann-Whitney U test). A reduction in serum albumin levels (below 35 g/L), combined with elevated protein levels in bronchoalveolar lavage (BAL), serves as a predictor of poor outcomes in patients with acute respiratory distress syndrome (ARDS), as indicated by a p-value of less than 0.01 (ANOVA).

**Conclusions:**

Our findings indicate that protein and albumin levels in bronchoalveolar lavage (BAL) fluid are elevated in severe acute respiratory distress syndrome (ARDS) cases. This suggests that BAL can effectively evaluate protein levels and fractions, which could significantly assist in assessing damage to the alveolocapillary membrane. Additionally, the increased albumin levels in BAL, often accompanied by a decrease in serum albumin levels, may serve as a valuable indicator of compromised integrity of the alveolar-capillary membrane in ARDS, with potential implications for patient care.

## Introduction

Patients with Acute Respiratory Distress Syndrome (ARDS) are critically ill and require treatment in the intensive care unit (ICU). The most common cause of direct injury to the alveolar epithelium is pneumonia, accounting for 60% of cases. Primary and secondary ARDS differences include relatively preserved type I and type II pneumocytes, more severely damaged capillary endothelium, and more significant interstitial oedema in extrapulmonary causes. The pathophysiology of ARDS, mainly Diffuse Alveolar Damage (DAD), was initially described in autopsies conducted on first reported cases in 1967 by Ashbaugh and colleagues. DAD is defined by the presence of hyaline membranes, along with interstitial oedema, necrosis, cell proliferation, and subsequent fibrosis at later stages. Nearly fifty years after that initial report, a new classification known as the Berlin classification was adopted at the European Society of Intensive Care Medicine (ESICM) annual meeting held in Berlin in 2011. Patients were stratified based on the severity of hypoxia, explicitly using the PaO2/FiO2 respiratory ratio, into mild, moderately severe, and severe subtypes of ARDS [1,2,3,4,5,6,7,8,9]. Alongside our efforts to treat COVID-19 ARDS (2020–2023 pandemia period), we also cared for a critically ill patient suffering from Influenza type A ARDS. We are investigating potential connections between these two distinctly caused ARDS cases.

The primary objective of this study is to demonstrate the presence of damage to the alveocapillary membrane in critically ill patients with COVID-19 and Influenza type A ARDS. We will assess the protein and albumin levels in bronchoalveolar fluid (BAL) and serum. Additionally, our secondary aim is to evaluate patient outcomes—specifically, survival or mortality—corresponding to elevated protein and albumin levels in BAL and serum while considering factors such as patient age and the duration of mechanical ventilation for those affected by COVID-19 and Influenza type A ARDS.

## Materials and Methods

A prospective, double-centre observational study was conducted across two intensive care units located in separate hospital centres: the University Clinical Center of the Republic of Srpska (Clinic for Intensive Care Medicine for Non-Surgical Patients) and the General Hospital Gradiska (Department of Anesthesia and Intensive Care Unit) in Bosnia and Herzegovina. The University Clinical Center of the Republic of Srpska ethics committee approved the research, with the approval numbers 01-19-110-2 / 21 and 01-1709-2 / 19. Patients were categorised into three distinct groups. The first group, designated as the COVID-19 group, consists of 30 individuals diagnosed with COVID-19, all presenting clinical signs of acute respiratory distress syndrome (ARDS) during the period from November to March 2021. The second group, known as the H1N1 group, comprises 14 patients with Influenza Type A (H1N1) who also exhibited clinical signs of ARDS and were treated from February to March 2019. Lastly, the control group includes 20 patients who did not display any lung damage and underwent elective one-day surgical procedures (hernia repair) under general endotracheal anaesthesia between February and March 2021. Inclusion criteria for subjects with viral pneumonia are based on the presence of PCR (polymerase chain reaction) confirmed causative agents, specifically Influenza type A (H1N1) and COVID-19 (SARSCoV-2). The control group must exhibit normal PCR sample findings.

The diagnosis of acute respiratory distress syndrome (ARDS) was determined following the Berlin Classification of Diseases. Our patients with ARDS met the criteria for a PaO2/FiO2 ratio of less than 200 mmHg, indicating moderately severe to severe ARDS, necessitating invasive mechanical ventilation.

Exclusion criteria for subjects included individuals younger than 18 years or older than 85 years, those with malignant diseases, liver failure, malnutrition, lung sarcoidosis, or heart failure as a cause of pulmonary oedema.

### Sampling and storage of samples procedure

The biomedical sampling procedure for our research is a standard method of taking blood and bronchoalveolar lavage fluid samples for laboratory diagnosis. Patients had blood samples, i.e. serum, taken before parenteral infusion of albumin solution. Bronchoalveolar lavage fluid was taken from patients with ARDS within the first 24 hours of intubation (and from the control group immediately after intubation for an elective surgical procedure) into sterile chambers via the external suction system. Bronchoalveolar lavage fluid was diluted with 10 ml of a 0.9% physiological solution and stored in a freezer at −20°C until laboratory analysis.

### Laboratory analysis

The laboratory analysis of serum albumin was conducted using an in vitro assay to quantitatively determine albumin levels in human urine, serum, plasma, and cerebrospinal fluid. For protein analysis in bronchoalveolar lavage fluid, we employed the Alinity c Protein Reagent Kit on an Alinity c analyser manufactured by Abbott^®^. This protein test utilises a turbidimetric procedure that involves benzethonium chloride as a protein denaturing agent. The proteins in the sample are denatured by benzethonium chloride, resulting in a suspension that is then quantified turbidimetrically at a wavelength of 404 nm. Albumin analysis in bronchoalveolar lavage fluid was performed using the Alinity c Microalbumin Reagent Kit on an Alinity c analyser manufactured by Abbott^®^. This microalbumin test is a turbidimetric immunochemical assay that employs polyclonal antibodies specific to human albumin. After mixing the sample, it binds with goat antibodies in the reagent, forming insoluble aggregates that increase the turbidity of the solution. The turbidity level is directly proportional to the albumin concentration in the sample and can be quantified using optical instruments.

### Statistical analysis

Statistical analysis was conducted using IBM SPSS Statistics for Windows, Version 25.0 (Released 2017; IBM Corp., Armonk, New York, United States). For continuous data, we used frequency, mean ± standard deviation, or median (interquartile range) as applicable. Categorical data were compared using the Chi-Square test. To assess the correlation between variables, we employed Pearson's Correlation test. The least significant differences between the three groups were analysed using Analysis of Variance (ANOVA) with Least Significant Difference (LSD) post hoc testing.

## Results

The mean ± standard deviation (SD) of albumin and protein values by groups confirmed that albumin levels in bronchoalveolar lavage fluid (BAL) and serum, along with total BAL protein values, were significantly higher in patients with lung damage compared to those with healthy lungs in the Control group (p < 0.001) – see Table 1.

**Table 1.** Proteins and albumins values between the patient group and outcomes

	**COVID-19 group (mean±SD)**	**H1N1 group (mean±SD)**	**Control group (mean±SD)**	p^*^	**Survivors (mean±SD)**	**Deceased (mean±SD)**	p^**^
BAL albumin (g/L)	1.63±1.35	1.55±1.46	0.07±0.07	<0.001	0.58±1.11	1.71±1.34	<0.001
BAL total protein (g/L)	1.53±0.71	1.47±0.71	0.43±0.35	<0.001	0.85±0.71	1.52±0.72	<0.001
Serum albumin (g/L)	28.73±4.97	31.93±4.85	38.43±4.03	<0.001	36.09±5.17	28.61±4.89	<0.001

* ANOVA LSD – the mean difference is Sig. at the 0.05 level;

** Independent Samples t-test – CI 95%; Sig. At the 0.05 level

The two patient groups, COVID-19 and H1N1, showed no significant difference in mean BAL albumin values (1.63 ± 1.35 vs. 1.55 ± 1.46; mean ± SD), with a p-value of 0.73, as determined by an independent t-test. Similarly, there was no significant difference in total proteins in BAL (1.53 ± 0.71; mean ± SD) with a p-value of 0.80, also from an independent t-test. However, there was a significant difference in mean serum albumin values between the COVID-19 group and the H1N1 group of ARDS patients on mechanical ventilation, with values of 28.73 ± 4.97 vs 31.93 ± 4.85 (mean ± SD), resulting in a p-value of 0.05, as indicated by an independent t-test.

The mean values of albumin and protein (mean ± SD) related to the outcome were significantly different between survivors and deceased patients, with a p-value of <0.001 (see Table 1).

**Fig. 1.**
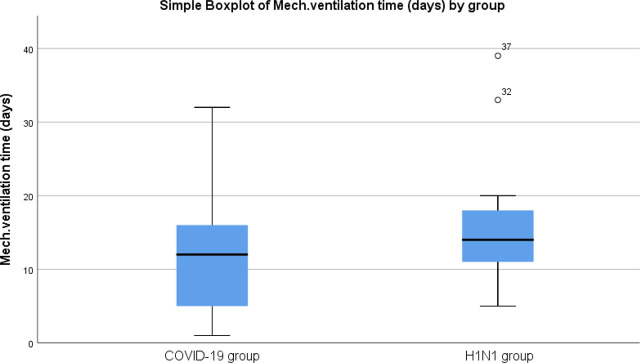
Mechanical ventilation days of ARDS (median, IQR) divided by groups

**Fig 2.**
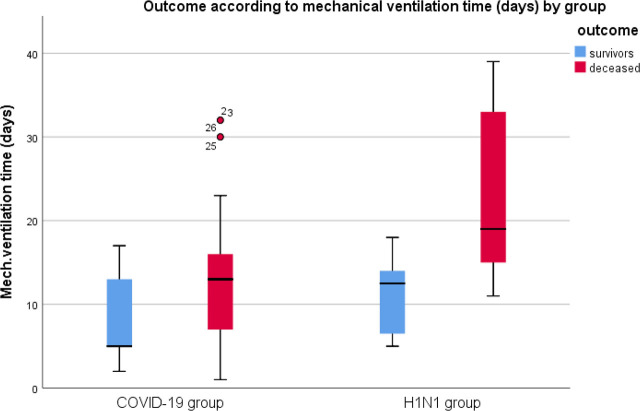
Comparison of ARDS patient outcomes between COVID-19 and H1N1 concerning mechanical ventilation duration.

In the H1N1 group, over half of the ARDS patients survived invasive mechanical ventilation, with a median duration of 14 days (interquartile range: 1–34). The COVID-19 group had a median duration of 13 days (interquartile range: 1–31) on mechanical ventilation. The survival rate in the COVID-19 group was significantly lower at 16.7%, compared to 57.1% in the H1N1 group, with a p-value of less than 0.01 (refer to Table 2, Figure 1, and Figure 2).

**Table 2.** Demographic variables, days of invasive mechanical ventilation and outcome between two groups of ARDS patients

	**COVID-19 group**	**H1N1 group**	p^*^	**Survivors**	**Deceased**	p^**^
Patients (n)	30	14	NA	13 (29.5%)	31(70.5%)	NA
Age (median, IQR)	72.5 (35–83)	62 (34–69)	<0.01	60 (35–70)	72 (34–83)	<0.01
Male (n,%)	17 (56.7%)	7(50%)	0.7	9 (37.5%)	15 (62.5%)	0.24
COPD (n,%)	3 (10%)	3 (21.4%)	0.43	1 (16.7%)	5 (83.3%)	0.57
CV diseases (n,%)	18 (60%)	6 (42.9%)	0.33	6 (25%)	18 (75%)	0.51
Diabetes (n,%)	6 (20%)	2 (14%)	0.71	1 (12.5%)	7 (87.5%)	0.35
CMV days (median, IQR)	13 (1–31)	14 (1–34)	0.27	13 (1–39)	13 (1–39)	0.5
Survivors (n,&)	5 (16.7%)	8 (57.1%)	<0.01	NA	NA	NA

COPD (chronic obstructive pulmonary disease); CV (cardiovascular); CMV (continuous mechanical ventilation).

* Mann-Whitney U test

** Pearson Chi-Square test

Additionally, the average age of patients in the COVID-19 group was notably older, with a median age of 72.5 years (range 35 – 83), compared to 62 years (range 34 – 68) in the H1N1 group (median, IQR), with a p-value of less than 0.01. This age difference may contribute to the unfavourable response to ARDS therapy (see Table 2).

## Discussion

Animal studies have shown that endothelial injury occurs a few minutes to hours after acute lung injury (ALI), resulting in intercellular endothelial cell gaps. Gaps in the endothelium allow the permeability of fluid, protein fractions and inflammatory factorsand contribute to further disruption of endothelial and epithelial barrier integrity [10].Such impaired alveolar-capillary membrane and the movement of fluids with proteins can help in the stratification of patients with ARDS via biomarkers in the bronchoalveolar lavage fluid. Biomarkers with the highest BAL values in the most severe forms of ARDS in reported studies and subsequent meta-analyses are proteins and albumins, which coincides with our findings in COVID-19 and Influenza type A (H1N1) ARDS patients [11,12,13,15]. On the other hand, metanalysis (49 studies) did not determine heterogeneity for individual markers because 2189 patients with different etiologies of ARDS were examined [16]. Our results confirm that elevated proteins and albumins in BAL and serum prove alveocapillary membrane damage in ARDS patients compared to patients with healthy lungs. A significant decrease in serum albumin in our sample also confirms that hypoalbuminemia is associated with mortality. Still, it is not known whether it is associated with complications, whereas hypoproteinemia is associated with ARDS in septic patients, as well as water retention in the body [16]. In our results, and from our experiences, the reason for the differences in serum albumin between the COVID-19 and H1N1 groups lies in the fact that the patient with Influenza type A (H1N1) ARDS required mechanical ventilation earlier in the onset of the disease than the patient with COVID-19. The serum albumin decreased less than in COVID-19 patients, where the average time from the disease onset to indications for invasive mechanical ventilation was longer.

The study by Hoeboer et al. concludes that the value of serum albumin below 20 g/L is more significant for the prediction and monitoring of the severity of ARDS more important than the value of C reactive protein in serum [17].

Acute lung injury - ALI and ARDS mainly affect the elderly population; for COVID patients, the arithmetic mean of the ages in our sample is a decade older. We should take into consideration that a state of the epidemic was not declared in Bosnia and Herzegovina in the case of the seasonal strain of the H1N1 influenza type A virus in 2019, while in the period February-March 2021, there was the third wave of the SARS-CoV 2 (COVID-19) coronavirus pandemic, so we can assume that we had a much higher influx of patients with severe clinical subtypes of viral pneumonia in COVID-19. A Chinese meta-analysis of the median age of COVID-19 patients with the most severe complications in terms of ARDS reports patients with a median of 67 and 70 years in the third wave of the pandemic, while in Europe, it is 62.5 years [10,18,19,20]. The Chinese study also has a decade younger group of patients with ARDS during Influenza type A (H1N1) pneumonia than COVID-19 ARDS [21].

Our results show that males are equally represented in relation to women in both the COVID-19 group and the H1N1 group, as other studies for H1N1 and COVID-19 patients also show [20,21,22,23].

Among our subjects with ARDS, the highest prevalence among comorbidities were cardiovascular diseases compared to type 2 diabetes or chronic obstructive pulmonary disease. In other reports, cardiovascular chronic diseases are the leading disease among patients with ALI and ARDS admitted to the intensive care unit, but also among those patients with a poor outcome [20,21,22,24].

Demographic research by *Sasson and Tang* indicates no statistical significance between patients with COVID-19 and H1N1 (from 2019); mortality was equal in both groups of patients [21,25]. Our results show a statistically significant difference in mortality between the two groups on invasive mechanical ventilation. COVID-19 ARDS patients had a worse outcome.

### Limitations of the study

An invisible limitation of the study was the negligible amount of scientific research related to examining BAL as a standard procedure in patients with ARDS. The most important limitation is the absence of randomisation criteria due to the unpredictable seasonal spread of COVID-19 and Influenza type A(H1N1) and the small number of patients with Influenza type A (H1N1) flu who met the requirements and required mechanical ventilation.

## Conclusions

Bronchoalveolar lavage (BAL) fluid is a valuable sample for biochemical analysis, offering insights into the severity of lung damage during lung injury. In patients with Acute Respiratory Distress Syndrome (ARDS), we can determine the severity of the condition by looking at protein and albumin levels in the BAL fluid. As serum albumin levels decline, BAL albumin levels rise in ARDS patients, which can be attributed to differences in the disease's progression from onset to requiring mechanical ventilation.
